# Sex and landscape influence spatial genetic variation in a large fossorial mammal, the Bare-nosed Wombat (*Vombatus ursinus*)

**DOI:** 10.1093/jmammal/gyae017

**Published:** 2024-03-27

**Authors:** Woei Jiun Tan, Scott Carver, Alynn M Martin, Nicholas M Fountain-Jones, Kirstin M Proft, Christopher P Burridge

**Affiliations:** Discipline of Biological Sciences, University of Tasmania, Private Bag 55, Hobart, TAS 7001, Australia; Discipline of Biological Sciences, University of Tasmania, Private Bag 55, Hobart, TAS 7001, Australia; Odum School of Ecology, University of Georgia, Athens, GA 30602, United States; Center for the Ecology of Infectious Diseases, University of Georgia, Athens, GA 30602, United States; Discipline of Biological Sciences, University of Tasmania, Private Bag 55, Hobart, TAS 7001, Australia; Caesar Kleberg Wildlife Research Institute, Texas A&M University–Kingsville, Kingsville, TX 78363, United States; Discipline of Biological Sciences, University of Tasmania, Private Bag 55, Hobart, TAS 7001, Australia; Discipline of Biological Sciences, University of Tasmania, Private Bag 55, Hobart, TAS 7001, Australia; Discipline of Biological Sciences, University of Tasmania, Private Bag 55, Hobart, TAS 7001, Australia

**Keywords:** burrow, fossorial, landscape genetics, marsupial, sex-biased dispersal

## Abstract

Dispersal is an important process that is widely studied across species, and it can be influenced by intrinsic and extrinsic factors. Intrinsic factors commonly assessed include the sex and age of individuals, while landscape features are frequently-tested extrinsic factors. Here, we investigated the effects of both sex and landscape composition and configuration on genetic distances among bare-nosed wombats (*Vombatus ursinus*)—one of the largest fossorial mammals in the world and subject to habitat fragmentation, threats from disease, and human persecution including culling as an agricultural pest. We analyzed a data set comprising 74 Tasmanian individuals (30 males and 44 females), genotyped for 9,064 single-nucleotide polymorphisms. We tested for sex-biased dispersal and the influence of landscape features on genetic distances including land use, water, vegetation, elevation, and topographic ruggedness. We detected significant female-biased dispersal, which may be related to females donating burrows to their offspring due to the energetic cost of excavation, given their large body sizes. Land use, waterbodies, and elevation appeared to be significant landscape predictors of genetic distance. Land use potentially reflects land clearing and persecution over the last 200 years. If our findings based on a limited sample size are valid, retention and restoration of nonanthropogenic landscapes in which wombats can move and burrow may be important for gene flow and maintenance of genetic diversity.

## Teaser

Female wombats appear to disperse farther than males, which may reflect mothers donating burrows to their offspring and moving away, as burrows are a costly resource for larger mammals. Agriculture areas appear more difficult for wombats to cross, perhaps reflecting past persecution.

Dispersal—the movement of an individual from its place of inception to its first place of reproduction or between successive reproductions (Danchin et al. 2008)—is an important component of life history which influences individual fitness, population demography, speciation, and extinction (Clobert et al. 2001; Garant et al. 2007). Therefore, our understanding of dispersal is crucial not only to comprehend evolution, but also for conservation scenarios, especially in habitats experiencing rapid environmental change (Aben et al. 2016). Dispersal can be quantified directly based on observations of individual movements and breeding success through time (Koenig et al. 2000; Webster et al. 2002; Berry et al. 2004), but because dispersal influences genetic differentiation between individuals and populations, can also be inferred from patterns of genetic variation (Bohonak 1999).

Dispersal can be influenced by extrinsic factors, such as landscape features (Kuefler et al. 2010; Vasudev et al. 2015). For example, the quality and availability of habitat can affect dispersal rates by increasing or decreasing the cost of movement (Travis and Dytham 1999). An increase in fragmentation of suitable habitat within the landscape generally decreases the propensity of an individual to disperse, resulting in heterogeneous dispersal patterns across the range of a species (Thomas 2000; Henriques-Silva et al. 2015). For instance, butterflies sampled in less fragmented landscapes were more likely to disperse than those sampled in more fragmented landscapes (Merckx et al. 2003). However, vagile species may be less affected by habitat fragmentation than more sedentary species or habitat specialists (Amos et al. 2012), and could actually disperse farther across fragmented than nonfragmented landscapes (Coulon et al. 2010; Spear et al. 2010; Berkman et al. 2013). The effects of landscape on dispersal are now commonly investigated by integrating genetic proxies for dispersal within landscape ecology—“landscape genetics” (Manel et al. 2003; Balkenhol et al. 2015).

Intrinsic factors such as sex, age, and body condition can also influence individual dispersal (Bowler and Benton 2005). In particular, sex-biased dispersal is prevalent in mammals and birds, and can arise from sex-specific strategies to increase fitness (Greenwood 1980). The exact causes of sex-biased dispersal are largely debated, but include mating systems, inbreeding avoidance, resource competition, and dominance interactions (Christian 1970; Greenwood 1980; Packer 1985; Perrin and Mazalov 2000). For example, competition for sex-specific resources such as breeding sites or mating partners can incentivize the sex facing intense competition to disperse farther (Perrin and Mazalov 2000). Individuals in different developmental stages may also experience different constraints on dispersal or pressure to disperse, such as having to acquire a new territory on maturation (Bowler and Benton 2005). For instance, dispersal in young female superb fairy-wrens (*Malurus cyaneus*) is mainly voluntary, while adult females disperse due to maternal aggression (Mulder 1995).

The Bare-nosed Wombat (*Vombatus ursinus*) is a large (77 to 115 cm, 17 to 39 kg), solitary, herbivorous, fossorial marsupial, distributed across southeastern mainland Australia, Flinders Island, and Tasmania (Triggs 2009; Carver et al. 2024). The species occurs from sea level to subalpine shrub/herblands, and generally spends 18 to 20 h per day in subterranean burrows, which serve as refugia from adverse environmental conditions (particularly daytime temperatures >20 to 25 °C) and potential predators (Triggs 2009; Carver et al. in press). Although not of formal conservation concern (Carver et al. 2021), and indeed also widely referred to as the “Common Wombat,” they have experienced localized disease-induced declines and extirpations, suffer high road mortalities, and can also be threatened by exotic predators, and human persecution by largely being culled as an agricultural pest (Martin et al. 2018; Thorley and Old 2020; Carver et al. 2021; Driessen et al. 2022a, 2022b). Therefore, knowledge of factors influencing dispersal is desirable for wombat conservation, which is encompassed by the Nature Conservation Act 2002 in Tasmania. Although a previous genetic study revealed that geographic distance explained genetic distance among Tasmanian individuals (Martin et al. 2019), it did not assess other extrinsic or intrinsic factors.

Habitat represents a key extrinsic factor that may influence *V. ursinus* dispersal, as the species prefers open grassland with shrub cover associated with riparian zones, avoiding rocky areas and areas of dense leafy vegetation (Roger et al. 2007; Evans 2008; Borchard et al. 2009). With respect to Tasmanian vegetation, sightings suggest that wombats are more common in agricultural and native grasslands and dry eucalypt forest and woodland, and rarer in wet eucalypt forest and rainforest (Driessen et al. 2022b). Roads pose a significant mortality risk, but may also promote dispersal in areas covered by dense vegetation (Roger and Ramp 2009; Crook et al. 2013). Observations suggest that dispersal by *V. ursinus* may be greater at higher elevations, potentially reflecting lower productivity and wombat density, the latter affecting distances required to find burrows (a shared resource) and mates (Matthews and Green 2012). However, areas of high topographic complexity (‘ruggedness’) may also impede *V. ursinus* dispersal given their ponderosity. Waterbodies are likely barriers to dispersal as wombats only swim occasionally (Borchard et al. 2009; Murray 2011). Intrinsic factors may also be important, and previous studies suggest female-biased dispersal in 2 wombat species (Johnson and Crossman 1991; Banks et al. 2002; Walker et al. 2008b). This contrasts to the more common male-biased dispersal in mammals (Greenwood 1980) and is hypothesized to reflect the energetic cost of burrowing in large animals, and the donation of burrows by female wombats to their offspring (Johnson and Crossman 1991). In this context, wombats (additionally comprising *Lasiorhinus latifrons*, *L. krefftii*) are one of the largest extant fossorial herbivorous mammals in the world (up to 50 kg; Triggs 2009).

Although studies investigating the determinants of dispersal are common, comparatively few assess both extrinsic and intrinsic factors (Oklander et al. 2010; Harrisson et al. 2013). In this study we tested whether sex and landscape factors influence dispersal in Tasmanian *V. ursinus* based on 30 males and 44 females genotyped for 9,064 single-nucleotide polymorphisms (SNPs). We hypothesize that dispersal is female-biased, consistent with previous observations of wombats and potentially related to energetic cost of burrowing by large animals. Therefore, we expect lower genetic differences among females than males across equivalent geographic distances. We also hypothesize lower genetic differences when sampling locations are separated by landscapes frequently exploited by wombats, rather than those features they avoid or in which they may face increased mortality.

## Materials and methods

### Samples, genotyping, and sexing

**Fig. 1.**
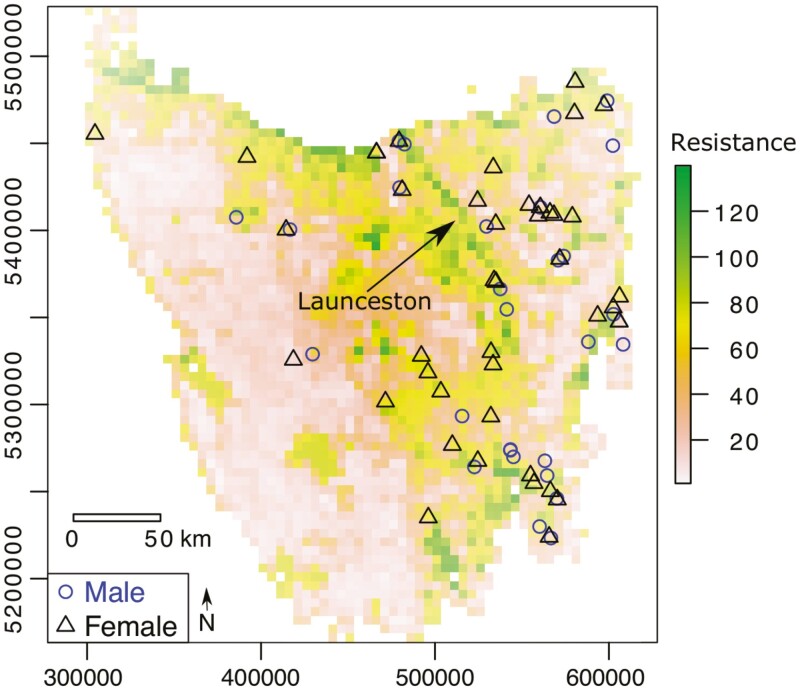
Location of *Vombatus ursinus* samples across Tasmania. Background represents the optimized resistance surface based on land use, waterbodies, and elevation.

This study analyzed an existing 9,064 SNP data set derived for 74 *V. ursinus* opportunistically sampled across its Tasmanian range during 2014 to 2017 by Martin et al. (2019; Fig. 1). The data set originally included 102 additional individuals sampled from mainland Australia and several islands, and was used to assess their genetic distinction from one another (Martin et al. 2019), but herein we restrict our landscape genetics analysis to mainland Tasmania given the highest sampling density. Genotyping was performed by Diversity Arrays Technology Pty Ltd (DArT), Canberra, Australia, for DArTseq analysis. DArTseq utilizes complexity reduction (restriction enzymes PstI and compliment, retained by DArT) and next-generation sequencing methodologies to produce genome-wide SNPs (Kilian et al. 2012). SNP filtering performed by Martin et al. (2019) included Hardy–Weinberg equilibrium, which should also remove any sex-linked markers. Using the R package *adegenet* (Jombart 2008), a pairwise genetic similarity matrix among individuals was generated as the proportion of shared alleles, *D*_ps_ (Bowcock et al. 1994). For the 21 wombats that had not been sexed based on anatomy, polymerase chain reaction (PCR) of the X-linked *G6PD* gene (primers: GpdEx12 ATTGCCTACGTCTATGGCAG, GpdEx13R CCACTTGTAGGTGCCCTCATACTGGAA; 175 bp) and the Y-linked *SRY* gene (primers: VursSRY152F TTCGTTGATGAGGCCAAACG, VursSRY152R TGTGTTAGGTGTTCGTGCTG, 152 bp; GenBank accession XM_027837454) were performed separately. Amplification of *G6PD* followed Sloane et al. (2000). PCR of the *SRY* gene used the same conditions except that the annealing temperature was 55 °C. In total, we analyzed 30 males and 44 females.

### Sex-biased dispersal

We tested for sex-biased dispersal using 2 approaches, generalized additive models (GAMs) and spatial autocorrelation. GAM was performed based on *D*_ps_, using the R package *mgcv* (Wood 2017). *D*_ps_ between individuals of the dispersing sex is expected to be higher than that for the philopatric sex at large geographic distances. Spatial autocorrelation analyses followed the approach of Banks and Peakall (2012); we compared 95% bootstrap confidence intervals around autocorrelation *r* values, and nonparametric heterogeneity tests (Smouse et al. 2008) were conducted at specific distance classes (squared paired-sample *t*-test statistic; *t*^2^) and across the entire correlogram (ω), with α set to 0.01. Specific distance classes were 10, 15, and 20 km. Spatial autocorrelation was performed with GenAlEx b.5 (Peakall and Smouse 2012) using 1,000 permutations and bootstrap replicates. Six even distance classes were employed under 3 different size intervals (10, 15, and 20 km) chosen to ensure approximately 20 observations within each distance class. Genotypic and geographic distances were calculated using GenAlEx. Given constraints on spreadsheet size in GenAlEx we analyzed 8,189 loci.

### Landscape genetics

Five extrinsic variables were hypothesized to influence *V. ursinus* dispersal: land use, water bodies (lakes, rivers, estuaries, watercourse, sea), vegetation, topographic ruggedness (roughness), and elevation. We conducted landscape genetics at a spatial resolution of 5,000 m, representing approximately 12 times the spatial extent of *V. ursinus* home range (18 ha on average; Evans 2008), given our widespread sampling and focus on broadscale landscape features. Similarly, observed distances traveled by *V. ursinus* can be up to 6.5 km in 1 night (Matthews and Green 2012), 25 km in other wombat species (Walker et al. 2008a), and *V. ursinus* home ranges may also be larger at higher elevations (~172 ha; Matthews and Green 2012). Individuals were also rarely represented by the same cell at this spatial resolution. Landscape rasters were initially generated at 50-m resolution in QGIS 3.4 (QGIS Development Team 2019) using already defined data from Land Information System Tasmania (LISTdata; https://www.thelist.tas.gov.au/app/content/data). Areas with Australian Land Use and Management (ABARES 2016) Primary Classification of 1 and 2 were reclassified as ‘natural,’ 3 to 5 as ‘anthropogenic,’ and waterbodies and wetlands were removed. A separate waterbodies raster scored as deep (1), shallow (2; potentially permeable to wombats at times, such as wetlands), and land (3). Vegetation was categorized according to the 11 classifications in TASVEG 3.0. Topographic ruggedness was estimated through the measurement of vectors orthogonal to the terrain surface—the vector ruggedness measure (Sappington et al. 2007)—based on a 25-m digital elevation model, that was also used separately to represent elevation. A terrestrially uniform raster was also used to represent geographic distance. Rasters were aggregated to 5,000-m resolution: means were used for land use, waterbodies, ruggedness, and elevation, and mode for vegetation.

Landscape genetics analyses were performed using *resistanceGA* (Peterman 2018), with 1 − *D*_ps_ as the response variable. Resistance distances were optimized using a genetic algorithm and across all 8 offered transformations of predictor variables (monomolecular and ricker transformations including ‘reverse,’ ‘inverse,’ and ‘inverse–reverse’ variants). Resistance distances were computed using the Julia implementation of Circuitscape (Hall et al. 2021) within *resistanceGA*. Circuitscape more realistically encapsulates the dispersal behavior of individuals (McRae et al. 2008; Pinto and Keitt 2009; Howey 2011). Optimizations were performed on all possible sets of predictors ('all_comb'), with effective distances estimated simultaneously for multiple predictors, rather than independently for each predictor prior to model fitting (Peterman and Pope 2021). Models were fit under a linear mixed-effects framework estimated via maximum likelihood population effects (MLPEs; Clarke et al. 2002; Van Strien et al. 2012). The best model was chosen among alternatives based on AICc (Shirk et al. 2018). The level of support for a resistance surface was assessed by bootstrapping the optimized distances (without replacement) 1,000 times and re-fitting the MLPE model and observing the frequency at which alternative models are inferred. The overall analysis was repeated 3 times with different random number seeds to avoid retention of a local search optimum.

## Results

### Sex-biased dispersal

**Fig. 2.**
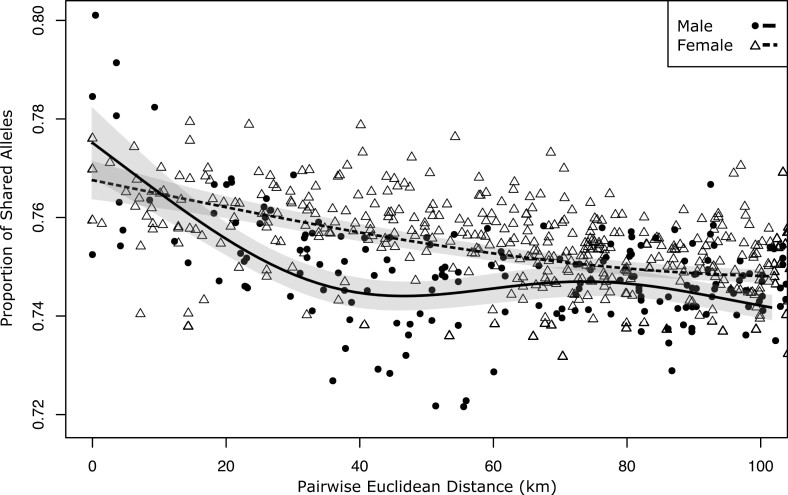
Generalized additive model predictions of mean (line) and 95% confidence interval (shaded) proportion of shared alleles based on pairwise geographic distance between *Vombatus ursinus* individuals of the same sex. Observed values are indicated by circles.

Evidence of female-biased dispersal was found in the Tasmanian *V. ursinus* population. Based on the GAM, the proportion of shared alleles declined significantly over geographic distance for all individuals (F=45.32, 
 
P<0.001), but females shared a higher proportion of alleles at almost all geographic distances than males (F=50.59, 
P<0.001; Fig. 2), consistent with female-biased dispersal. However, spatial autocorrelation could not reject unbiased dispersal across the 3 distance intervals trialed (10, 15, and 20 km) as bootstrap confidence intervals for the *r* coefficient (autocorrelation) were overlapping for the sexes and *t*^2^ tests at each distance class, and the ω test (heterogeneity test) across entire correlograms, were not statistically significant (*P* > 0.01). However, the correlograms are suggestive of female-biased dispersal, as males exhibited higher spatial autocorrelation at short geographic distances than females (0 to 10 km; Supplementary Data SD1).

### Landscape factors.

The model containing land use, water bodies, and elevation offered the best prediction of 1 − *D*_ps_ (Fig. 1; Table 1). Given the transformations selected, increasing genetic distance is related to greater prevalence of anthropogenic land (monomolecular transformation, with natural habitats having smaller values), lower prevalence of challenging water bodies (reverse monomolecular transformation—deep water = 1, shallow = 2, land = 3), and higher elevation (inverse–reverse monomolecular). Within this model, the land use raster contributed 44% to the final composite resistance surface, water bodies 45%, and elevation 11%. However, land use appeared in the 11 highest-ranked models, was also the highest-ranked single surface optimization, and the third most frequent best model from bootstrap replicates (17%, vs. 35% for land use + water bodies + elevation, and 23% for land use + waterbodies). Topographic ruggedness did not feature in models within 10 AICc of the best model. Two replicate runs returned the same 2 top models (<4.0 ΔAICc) and associated inferences, except that the land use model received much lower bootstrap support in 1 instance (2%; Supplementary Data SD2).

**Table 1.** Linear mixed-effects models for environmental variables on genetic distance (1 − *D*_ps_) in *Vombatus ursinus* from Tasmania. Only the top 11 models are shown. *k* is number of parameters, which for continuous surfaces is a single intercept plus the transformation, shape, and maximum value for each surface, and for each categorical surface the number of categories. *R*^2^_m_ is marginal *r*^2^.

Model	*k*	*R* ^2^_m_	AICc	ΔAICc	AICc weight	Bootstrap % top model
Land use, waterbodies, elevation	10	0.47	−20,507.45	0.00	0.84	35.1
Land use, waterbodies	7	0.49	−20,504.11	3.34	0.16	23.8
Land use, waterbodies, ruggedness	10	0.49	−20,495.67	11.78	0.00	0.0
Land use, elevation, ruggedness	10	0.47	−20,489.18	18.27	0.00	2.7
Land use, ruggedness	7	0.49	−20,486.40	21.05	0.00	3.5
Land use, waterbodies, elevation, ruggedness	13	0.46	−20,486.32	21.14	0.00	0.0
Land use	4	0.42	−20,483.24	24.21	0.00	17.4
Land use, waterbodies, vegetation	17	0.53	−20,480.90	26.55	0.00	0.2
Land use, ruggedness, vegetation	17	0.49	−20,471.75	35.70	0.00	0.0
Land use, elevation	7	0.43	−20,470.29	37.17	0.00	0.0
Land use, waterbodies, elevation, vegetation	20	0.49	−20,464.60	42.86	0.00	0.0

## Discussion

### Sex-biased dispersal

We observed a significant female bias in dispersal in *V. ursinus* based on GAM. Given that sex-biased dispersal should only be apparent over spatial scales reflecting single generational movement, the GAM curves suggest that single-generation dispersal by female *V. ursinus* in our study range could be in the order of 50 km or more. However, inferences from spatial autocorrelation were not significant, and larger sample sizes should be used to further test the presence and spatial scales of sex-biased dispersal.

While female-biased dispersal is unusual for mammals (Greenwood 1980), its presence in all 3 extant wombat species and in different populations (Johnson and Crossman 1991; Banks et al. 2002; Walker et al. 2008b) suggests an important underlying ecological and evolutionary basis. Female-biased dispersal in these species could be a consequence of obligate maternal care and the energetic demands of digging a burrow, with adult females donating their burrows to their offspring (Johnson and Crossman 1991). Burrows are central to the life history of all wombat species and can generally be categorized by length, with burrows >5 m being the most frequently occupied, and maintained by multiple wombats, and likely across generations (McIlroy 1973). In contrast, male wombats exhibit a much lower propensity to disperse, remaining near their natal burrows and occasionally sharing burrows with close male kin (Taylor et al. 1997; Walker et al. 2008b).

This is a pattern distinct from other burrowing mammals such as the Burrowing Bettong (*Bettongia lesueur*), which exhibit male-biased dispersal, potentially due to intraspecific aggression in males (Parsons et al. 2002). The energetic demands associated with burrow establishment in the burrowing bettongs are also likely significantly less due to their smaller body size (1.3 kg vs. wombats up to 50 kg) and greater burrow sharing (Parsons et al. 2002). Body size also appears to be an important predictor of sex-biased dispersal in fossorial eutherian mammals. Male-biased dispersal is observed in small species, such as Common Vole (*Microtus arvalis*; Gauffre et al. 2009) and Fisher (*Pekania pennanti*; Tucker et al. 2017). In contrast, larger fossorial eutherians such as the European Badger (*Meles meles*) can exhibit female-biased dispersal, although it appears to be context-dependent (Gaughran et al. 2019). Indeed, substrate type has been observed to influence sex-biased dispersal within Southern Hairy-nosed Wombat (*L. latifrons*), perhaps through altered cost of burrowing (Walker et al. 2007).

### Land use and water

While a significant isolation-by-distance relationship is evident for Tasmanian *V. ursinus* (Martin et al. 2019), from the current study it is apparent that the nature of intervening landscape is more influential than geographic distance alone (null model ΔAICc = 64.97). Land use appeared to be the most important predictor of genetic distance in *V. ursinus* based on its appearance in the 11 best models and contribution to composite resistance surfaces. Following colonization by the British in 1803, Tasmania experienced a dramatic change in anthropogenic modification to the landscape. Vast areas of central Tasmania were converted to grazing of introduced mammals (Michaels et al. 2010). While wombats actually appear disproportionally abundant in agricultural landscapes (Taylor 1993; Skerratt et al. 2004; Roger et al. 2007; Evans 2008; Borchard et al. 2009), high-quality habitat could also act to reduce the need for movement, with potential consequences for gene flow (Spear et al. 2010). Additionally or alternatively, wombats likely experienced substantial persecution from the British in central Tasmania owing to perceived competition with livestock and damage to infrastructure such as fences (Thorley and Old 2020). Wombats were extirpated from several Bass Strait islands by the British for similar reasons (Rounsevell et al. 1991). Furthermore, crop protection permits have until recently been issued frequently for the culling of wombats (Carver et al. 2021). Between 1996 and 2016 this represented an average annual quota of 639 individuals (range = 220 to 2,205), but the actual number harvested cannot be ascertained accurately based on permit reports, and unlicensed culling also needs to be considered (Carver et al. 2021). Earlier land management by indigenous peoples—burning to maintain grasslands—may have also reduced wombat gene flow in central Tasmania, but historical records suggest that hunting is unlikely to have been influential (Cosgrove 1984).

Wombats are less commonly encountered near contemporary high-density human habitations in Tasmania (Driessen et al. 2022b), and hence the city of Launceston, located at the head of the Tamar Estuary, may have contributed to the elevated genetic distances observed across this region (Fig. 1). This estuary itself is also likely important, given that water appears to be positively associated with increasing genetic distance in *V. ursinus* and the Tamar is one of the largest waterways within the study region. While *V. ursinus* has been observed to swim (Borchard et al. 2009; Murray 2011), it is generally over distances less than 10 m.

### Elevation

Although elevation featured in some of the better models predicting genetic distance, the nature of the selected transformation (inverse–reverse monomolecular) invokes higher resistance at higher elevations. This pattern conflicts with telemetry observations that *V. ursinus* at higher elevations on mainland Australia move farther and have larger home ranges, perhaps reflecting lower wombat densities and the distances required to access burrows, mates, and forage (Matthews and Green 2012). However, movements undertaken by individuals within established home ranges may be decoupled from those representing gene flow (natal and breeding dispersal; Ronce 2007) in which animals may be bolder and more likely to traverse areas of lower suitability (Trainor et al. 2013; Keeley et al. 2016). Furthermore, differences in wombat density with elevation have not been established for Tasmania, although they occupy all elevations (Driessen et al. 2022b). In the Tasmanian landscape topographic ruggedness is largely decoupled from elevation given that an expansive high-elevation plateau exists (the Central Plateau), and hence elevation is not explaining gene distance via ruggedness. The first model to include ruggedness had ΔAICc = 11.78.

### Roads

Because roads are strongly correlated with other anthropogenic features across our landscape, they were combined in our single land use surface. However, road-induced mortalities are high in some areas (Roger and Ramp 2009; Crook et al. 2013; Thorley and Old 2020; Driessen et al. 2022b), and the potential influence on gene flow would be best studied genetically where roads appear uncorrelated with adjacent landscape features (e.g., entirely within agricultural settings). We also caution that the nature of Tasmanian roads (rarely more than 2 lanes) contrasts strongly with other systems where they appear to be influential on gene flow (Kozakiewicz et al. 2019).

### Vegetation

The type of vegetation within the landscape did not have a strong influence on genetic distance in *V. ursinus*, despite reputed habitat preferences for native grassland and treeless areas, and dietary preference for monocots (Evans et al. 2006). However, habitat quality can have contrasting effects on gene flow—animals should move predominantly within better habitats, but may also not need to move as far to satisfy their resource requirements (Spear et al. 2010). The regions intervening our sampling points (Supplementary Data SD3) were typically of suitable vegetation associations noted for Tasmanian wombats, such as agricultural and native grasslands and dry eucalypt forest and woodland (Driessen et al. 2022b), which may have also influenced our results.

### Future studies

In our study we have provided evidence that dispersal in *V. ursinus* is influenced by both sex and landscape features. This raises the possibility that future studies consider whether landscape effects on *V. ursinus* dispersal are sex-specific. This possibility is rarely assessed despite many species exhibiting sex bias in habitat requirements and use (Conde et al. 2010; Harrisson et al. 2013), but benefits from sample sizes much larger than ours (e.g., Amos et al. 2014; Tucker et al. 2017; Zeller et al. 2023). Sex-specific landscape effects are already suggested for another wombat, where soil characteristics appear to influence the propensity of female-biased dispersal (Walker et al. 2008b).

The inferences of our study need to be framed in the context of its geographic sampling. This includes the number of individuals sampled, and their relative and absolute locations. For instance, the spatial scale of sampling can influence the ability to detect sex-biased dispersal (Banks and Peakall 2012). Likewise, outcomes of landscape genetic studies may also depend on their spatial resolution (Anderson et al. 2010). Wombats are challenging for direct sampling, with previous genetic investigations employing indirect hair sampling for microsatellite genotyping (Sloane et al. 2000; Banks et al. 2002; Walker et al. 2007, 2008a, 2008b), but such samples may experience lower performance during genomic approaches (Valencia et al. 2018). In this study, sampling was constrained by heavy reliance on roadkill specimens and preexisting material. Future studies that employ more samples obtained from different environments and at different spatial scales are therefore desirable to validate our inferences.

### Conservation implications.

Findings from this study can be used for conservation purposes such as habitat corridor or connectivity planning (Segelbacher et al. 2010; Proft et al. 2018). With respect to genetic diversity, emphasis should be placed on maintaining and restoring nonanthropogenic landscapes that provide connections among existing wombat populations and locations for wombats to burrow. Where significant barriers to wombat dispersal exist, assisted gene flow could be considered if reductions in genetic diversity are observed or anticipated in the future. Recent increases in wombat abundance, and declines in persecution (Carver et al. 2021; Driessen et al. 2022b) could also see a future increase in gene flow.

## Supplementary data

Supplementary data are available at *Journal of Mammalogy* online.

**Supplementary Data SD1.**—Correlogram for male and female *Vombatus ursinus* based on 6 even distance classes of 10-km interval. Error bars represent 95% confidence error bars determined by bootstrapping. Identical inferences were derived when using 15- and 20-km intervals (not shown).

**Supplementary Data SD2**.—Results from linear mixed-effects models for the effects of the environmental variables on genetic distance (1 – *D*_ps_) in *Vombatus ursinus* from Tasmania. Only the top 11 models based on AICc from the first replicate run are shown. “Bootstrap” represents the proportion of bootstrap replicates in which the model was best ranked.

**Supplementary Data SD3.**—Sampling locations of *Vombatus ursinus* relative to the 11 vegetation classes used for landscape genetics analyses.

gyae017_suppl_Supplementary_Datas_SD1

gyae017_suppl_Supplementary_Datas_SD2

gyae017_suppl_Supplementary_Datas_SD3

## Data Availability

Reference sequences and SNP genotypes are available at the Dryad Digital Repository: https://doi.org/10.5061/dryad.5t37q5f.
