# Supplementary material for: Sex and landscape influence spatial genetic variation in a large fossorial mammal, the Bare-nosed Wombat (Vombatus ursinus)
Source: J Mammal. 2024 Mar 27;105(3):481–9. doi: 10.1093/jmammal/gyae017 (PMC11130529; doi:10.1093/jmammal/gyae017)
Supplement: gyae017_suppl_Supplementary_Datas_SD1 [file gyae017_suppl_supplementary_datas_sd1.docx]

**Supplementary Data SD1.** Correlogram for male and female Vombatus ursinus based on six even distance classes of 10 km interval. Error bars represent 95% confidence error bars determined by bootstrapping. Identical inferences were derived when using 15 and 20 km intervals (not shown).
