# Supplementary material for: Sex and landscape influence spatial genetic variation in a large fossorial mammal, the Bare-nosed Wombat (Vombatus ursinus)
Source: J Mammal. 2024 Mar 27;105(3):481–9. doi: 10.1093/jmammal/gyae017 (PMC11130529; doi:10.1093/jmammal/gyae017)
Supplement: gyae017_suppl_Supplementary_Datas_SD2 [file gyae017_suppl_supplementary_datas_sd2.docx]

**Supplementary Data SD2.** Results from Linear Mixed Effects models for the effects of the environmental variables on genetic distance (1-*D_ps_*) in *Vombatus ursinus* from Tasmania. Only the top 11 models based on AICc from the first replicate run are shown. ‘Bootstrap’ represents the proportion of bootstrap replicates in which the model was best ranked.

|  | Replicate 1 | | | Replicate 2 | | | | Replicate 3 | | | |
| --- | --- | --- | --- | --- | --- | --- | --- | --- | --- | --- | --- |
| Model | ΔAICc Bootstrap | | | | ΔAICc Bootstrap | | | | ΔAICc Bootstrap | | |
| Land use, waterbodies, elevation | 0.0 | 35.1 | 0.0 | | | 47.4 | 1.1 | | | 16.4 |  |
| Land use, waterbodies | 3.3 | 23.8 | 2.7 | | | 32.0 | 0.0 | | | 37.8 |  |
| Land use, waterbodies, ruggedness | 11.8 | 0.0 | 11.1 | | | 0.0 | 9.8 | | | 0.3 |  |
| Land use, elevation, ruggedness | 18.3 | 2.7 | 21.1 | | | 0.0 | 21.3 | | | 4.1 |  |
| Land use, ruggedness | 21.1 | 3.5 | 21.4 | | | 1.0 | 18.3 | | | 0.2 |  |
| Land use, waterbodies, elevation, ruggedness | 21.1 | 0.0 | 15.3 | | | 0.0 | 7.4 | | | 6.4 |  |
| Land use | 24.2 | 17.4 | 55.5 | | | 2.3 | 21.1 | | | 20.3 |  |
| Land use, waterbodies, vegetation | 26.6 | 0.2 | 24.7 | | | 0.0 | 22.8 | | | 0.0 |  |
| Land use, ruggedness, vegetation | 35.7 | 0.0 | 55.7 | | | 0.0 | 34.8 | | | 0.0 |  |
| Land use, elevation | 37.2 | 0.0 | 36.9 | | | 0.4 | 33.6 | | | 0.0 |  |
| Land use, waterbodies, elevation, vegetation | 42.9 | 0.0 | 47.4 | | | 0.0 | 40.7 | | | 0.0 |  |
