# Supplementary material for: Sex and landscape influence spatial genetic variation in a large fossorial mammal, the Bare-nosed Wombat (Vombatus ursinus)
Source: J Mammal. 2024 Mar 27;105(3):481–9. doi: 10.1093/jmammal/gyae017 (PMC11130529; doi:10.1093/jmammal/gyae017)
Supplement: gyae017_suppl_Supplementary_Datas_SD3 [file gyae017_suppl_supplementary_datas_sd3.docx]

**Supplementary Data SD3.** Sampling locations of *Vombatus ursinus* relative to the 11 vegetation classes used for landscape genetics analyses.
